# Efficient Parallel Levenberg-Marquardt Model Fitting towards Real-Time Automated Parametric Imaging Microscopy

**DOI:** 10.1371/journal.pone.0076665

**Published:** 2013-10-10

**Authors:** Xiang Zhu, Dianwen Zhang

**Affiliations:** 1 College of Information and Electrical Engineering, China Agricultural University, Beijing, China; 2 College of Economics & Management, China Agricultural University, Beijing, China; 3 Imaging Technology group, Beckman Institute for Advanced Science & Technology, University of Illinois at Urbana-Champaign, Urbana, Illinois, United States of America; University of Adelaide, Australia

## Abstract

We present a fast, accurate and robust parallel Levenberg-Marquardt minimization optimizer, GPU-LMFit, which is implemented on graphics processing unit for high performance scalable parallel model fitting processing. GPU-LMFit can provide a dramatic speed-up in massive model fitting analyses to enable real-time automated pixel-wise parametric imaging microscopy. We demonstrate the performance of GPU-LMFit for the applications in superresolution localization microscopy and fluorescence lifetime imaging microscopy.

## Introduction

Biophysical and biomedical data often have to be fitted to known models to extract the parameters that are of interest, and quantitative parametric imaging techniques have been increasingly utilized. For example, in magnetic resonance imaging (MRI), the relaxation time images are created by fitting the measured magnetic resonance signal data of each pixel to theoretical relaxation models [1]; in superresolution localization microscopy (SRLM) or single-particle tracking microscopy, the positions of tens to hundreds of single particles need be determined for an image by generally fitting two-dimensional (2D) single particle images to the point spread function (PSF) of imaging system [2], [3]; and similarly, in fluorescence lifetime imaging microscopy (FLIM) using the time-correlated single-photon counting (TCSPC) technique, a widely used method to extract the fluorescence lifetime at each image pixel is a nonlinear model fitting method that involves iterative re-convolution of the instrument response function (IRF) and single or multiple exponential functions [4], [5]. For all these techniques, fast and accurate model fitting is often a crucial step towards real-time automated pixel-wise parametric imaging, because the calculation procedure of the model fitting has to be repeated tens of thousands of times for an image, and the total analysis tends to be rather slow.

In various scientific disciplines, the Levenberg-Marquardt (LM) method [6] has become a standard technique for nonlinear minimization problems, and it is widely adopted for dealing with model fitting applications. Although there have been a large number of minimization optimizers available now, they are often highly tuned for specific applications, and many of them have to been modified before they can be applied for a new fitting function [3], [4]. Among the large number of existing LM minimization optimizers, MPFit [7] was developed by Dr. Craig Markwardt (University of Wisconsin, WI) based on an early minimization routines library Minpack [8], and has been recently reported [1], [2], [9]–[20] as an efficient and robust optimizer for a variety of fitting functions in various applications and in many disciplines. MPFit was found to be most frequently used in astrophysical research, for example, to analyze the SOPHIE spectra observed from DI Herculis [9], and to derive the shape of the x-ray spectrum obtained from of Mercury’s Surface by the MESSENGER spacecraft [10]. In biophysical and biomedical science, MPFit has also often been used, for example, in the applications of magnetic resonance imaging [1], [11]–[15], optical particle tracking [2], [16], [17] and small-angle X-ray scattering [18], [19]. In addition, in environmental science, MPFit was reported to be used to analyze the normalized difference vegetation index time series data from satellite moderate resolution imaging spectroradiometer [20].

Motivated by the applications towards real-time automated pixel-wise parametric imaging microscopy, we applied parallel computing algorithms to MPFit. We have achieved a new LM minimization optimizer, which is implemented on graphics processing unit (GPU) and we call GPU-LMFit. The parallel computing model of GPU-LMFit follows NVIDIA (http://www.nvidia.com/) compute unified device architecture (CUDA). With GPU-LMFit, one LM data fitting procedure is completed in one CUDA block by tens to hundreds of threads running in parallel. A GPU device can allow multiple CUDA blocks and thus a number of LM model fittings executing concurrently to realize a dramatic increase in speed of massive model fittings analyses.

Here we demonstrate the performance of GPU-LMFit for the applications in SRLM and FLIM. Two software tools called GPU2DGaussFit and GPUFLIMFit have been made respectively for single-molecule localization analysis in SRLM and TCSPC histogram fitting analysis in FLIM by using GPU-LMFit implemented with a modified maximum likelihood estimator (MLE), because MLE is better in terms of precision than traditional least-squares estimator in the data fitting analysis in both SRLM [3] and FLIM [4]. The fitting precision is measured as the standard deviation of the estimates of the parameters resolved from the fittings in a simulation. The CUDA C libraries of GPU-LMFit, the source code of GPU2DGaussFit and the Matlab (The Mathworks, Natick, MA) simulation programs for the performance tests of both GPU2DGaussFit and GPUFLIMFit are available in File S1.

## Methods

### The Architecture of GPU-LMFit

For pixel-wise parametric imaging techniques using the LM method, the calculation procedure of a LM fitting algorithm is implemented for each image pixel, so it has to be repeated tens of thousands of times for the entire image, and thus the total analysis tends to be rather slow. To enable real-time automated parametric imaging microscopy, we developed a parallel LM minimization optimizer, GPU-LMFit, for high performance scalable processing of massive model fittings. Generally a parallel computer program is more difficult to write than a sequential one [21], because communication and synchronization between the different threads are typically some of the greatest obstacles to obtain good parallel program performance, and concurrency can introduce some new classes of potential software bugs, of which race conditions are the most common.

As with MPFit, GPU-LMFit is to find a vector solution 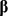 for the fitting parameters that give a local minimum to the sum 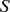 of squares of a user-defined fitting function 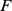:
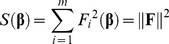
(1)where 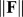 represents the Euclidean norm of the vector of the fitting function values. Here vectors and matrices are denoted by bold letters. In Equation (1), 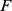 can be generally found in the form of
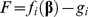
(2)for least-squares estimator (LSE). In Equation (2), 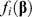 and 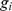 represent the model function and the experimental data, respectively. The subscript 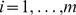 refers to the index of the data point, and 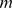 is the number of data points. Since it has been found [3], [4] that minimizing MLE for Poisson distributed data can yield better precision of the parameters determined in data fitting, we took the fitting function 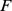 as 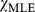 in Equation (6) to use the MLE derived by Laurence et al [4] for light microscopy data.

A standard LM algorithm [7] is used in MPFit and it is an iterative procedure and searches for the solution 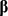 starting with an initial guess 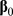. In the 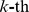 iteration step, 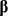 is updated by a new estimate 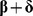, and the increment 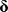 is determined by solving the equations [22], [23]

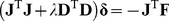
(3)where the scalar 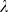 is the damping factor and controls both the magnitude and direction of 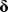; 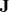 denotes Jacobian matrix of 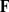 and the superscript T denotes matrix transpose; 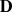 is the diagonal scaling matrix, which accounts for the problems from the poorly scaled models in which the fitting parameters differ by several orders of magnitude. The scaling matrix 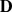 is determined by the diagonal elements of 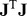. The default for the scaling matrix 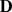 is the square root of the diagonal of 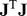. Equations (3) are just the normal equations for the linear least-squares minimization problem:



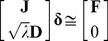
(4)Using the QR factorization 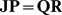, Equations (4) can be solved iteratively as
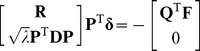
(5)where 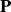, 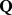 and 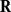 are the permutation matrix, the orthogonal matrix and the upper trapezoid matrix [23].

As discussed above, the LM algorithm has at least two main computational tasks, which are 1) the QR factorization of Jacobian matrix 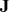 and 2) the determination of LM damping factor 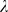. If there is not an analytical Jacobian available for the user-defined fitting function, a third computational task that must be performed is to compute an approximation to the Jacobian, and this is computed in MPFit using a forward-difference approach [8], in which many independent computations are involved and are very appropriate for parallel programming. The QR factorization is computed in MPFit by using Householder transformation [24]. Among all three abovementioned major computational tasks, we have found that the QR factorization of Jacobian usually dominates the total computational cost, and it can account for 60–80% of computational time of a fitting process, because it involves a large amount of matrix and vector operations, which are, however, also very appropriate for high-performance parallel linear algebra computation at which GPU technology excels.

**Figure 1:**
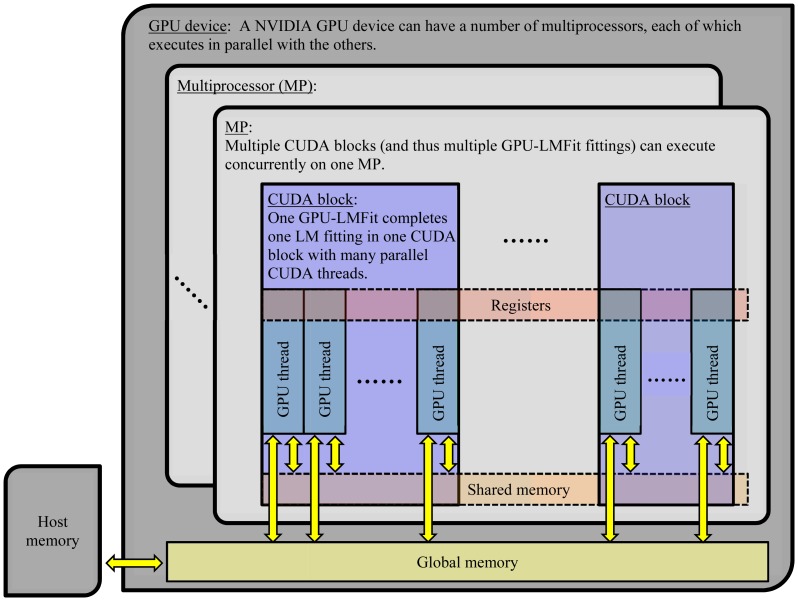
The architecture of massive parallel model fittings with GPU-LMFit. In pixel-wise parametric imaging applications, a large amount of experimental data for all image pixels are first collected and saved in host computer memory and then passed to the GPU global memory for efficient parallel model fitting analyses with GPU-LMFit using NVIDIA CUDA, which has a generic C-like language interface and it offers a data parallel programming model that is supported on NVIDIA GPUs. In CUDA, the host program launches kernel functions running on the GPU. A kernel function is organized as a hierarchy of threads. GPU Threads are grouped into blocks, and blocks are grouped into a grid. For each fitting, the parallel algorithms in GPU-LMFit, user-defined fitting function and/or Jacobian function are implemented in a single CUDA block by more than tens of GPU threads, which can synchronize and share data with each other and are all executed on a single GPU multiprocessor (MP). A GPU MP allows multiple GPU blocks running concurrently and a GPU device can have a number of GPU MPs, so using GPU-LMFit, a large number of LM model fittings can execute concurrently in a GPU, and each of the fittings is performed with parallel algorithms by many GPU threads in parallel.

**Figure 2:**
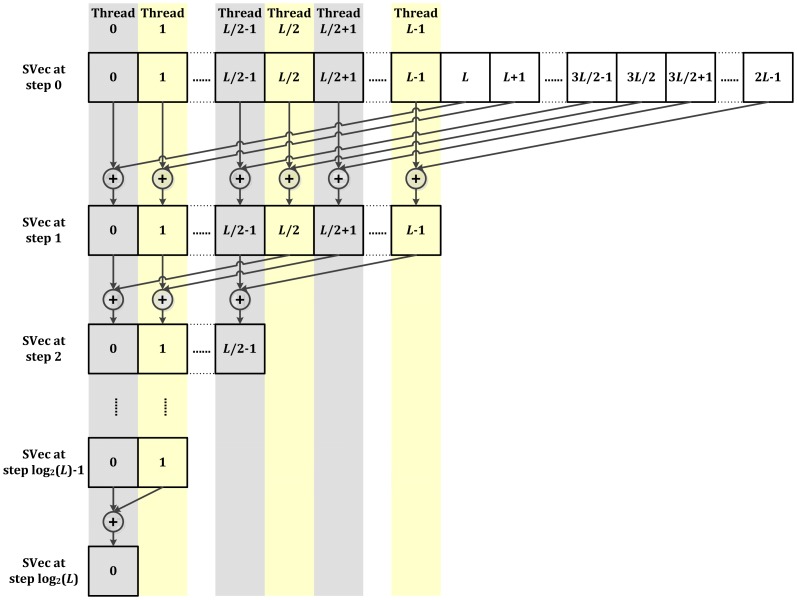
An example of scalable parallel reduction in GPU-LMFit: the computation of the Euclidean norm 
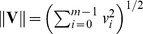
 of a vector 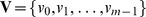. Scalable parallel reductions in GPU-LMFit need to be implemented by all threads of a CUDA block using GPU shared memory, and it also requires that the number 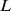 of total threads of the CUDA block must be power of 2, and the shared memory must be configured for the storage of at least 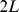 elements data. Here we assume that an array named 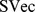 is created in the shared memory to store the intermediate results for scalable parallel reduction. The computational implementation consists of two phases: in the first phase, each thread first stepwisely calculates the sum of the squares of 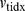 and every other 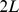 elements in the vector, and writes the results to 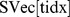, where 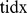 is the index (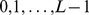) of the thread in the block and 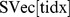 denotes 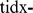 indexed element of 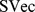. Next, each thread calculates the sum of the squares of 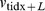 and every other 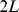 elements in the vector, and the results are written to 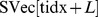, so after the first phase, each element of 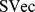 contains the partial sum 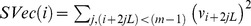 of the vector element squares; in the second phase, the parallel sum reduction is implemented to 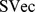 by all threads following the above algorithmic pattern (sum reduction tree). This sum reduction tree is not an actual data structure, but to show the concept we use to determine what each thread does at each step of the traversal. The numbers in the square leaves on the tree represent the index of element of 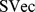. In every step, each thread updates the value of 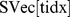 with two 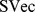 element connected with arrows, and all threads are synchronized at the end of each step, so that after 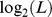 steps, the value of squares of 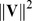 is obtained in 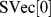. Therefore, using the scalable parallel reduction in GPU-LMFit, the computation of 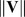 is accomplished through maximum 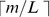 square operations, 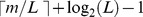 addition operations, and one square root operation per thread, where 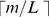 is the smallest integer not less than 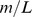.

GPU-LMFit uses a scalable parallel LM algorithm that we developed and optimized for using NVIDIA CUDA. Figure 1 presents the architecture of the implementation of GPU-LMFit on massive parallel model fittings. As shown in Figure 1, each fitting is processed by many GPU threads in parallel. Many codes in the sequential LM algorithm have been developed and optimized for GPU-LMFit to use the efficient scalable parallel reduction [25], which converts certain sequential computations for associative and commutative operations in a collection of data into equivalent, but parallel, computations and can finally reduce the data set to get a single value. Figure 2 presents a typical example for scalable parallel reduction, which is the computation of the Euclidean norm of a vector and used extensively in GPU-LMFit. For example, the computation of the Euclidean norm 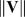 of a 200 elements vector 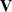 requires 200 square operations, 199 addition operations and one square root operations in a sequential computation. In GPU-LMFit, 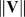 is computed by all CUDA threads of a CUDA block in parallel using the scalable parallel reduction. As described in Figure 2, if a CUDA block for GPU-LMFit is configured to have 128 threads, then the computation of 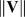 in GPU-LMFit will take only the maximum computation time in which a thread can complete 2 square operations, 8 addition operations and one square root operations. Therefore, the parallel LM algorithm that we developed in GPU-LMFit can be much more efficient for a single fitting than the traditional sequential algorithm. In addition, as in Figure 1, a number of fittings using GPU-LMFit can execute concurrently in a single GPU device, and this makes an ideal method for efficient automated massive parametric imaging techniques, where the fitting procedure has to been repeated independently for each image pixel to fit the measured data in the pixel to a numerical model to resolve the parameters that are of interest.

Another important feature of the architecture of GPU-LMFit in Figure 1 is that it can allow multiple GPUs applications, where the measured experimental data in the host computer memory is separately passed to the global memories of multiple GPUs, and then the host program launches the kernel functions on each GPU device. Therefore, the multiple GPUs application can further improve the efficiency of the LM model fittings with GPU-LMFit, and enable real time automated massive parametric analyses for the microscopy methods using new detector technologies with the rising rate of data output.

An obvious advantage of the use of GPU for real-time model fitting analyses in parametric imaging microscopy is that GPU-based computations can reduce the usage of central processing unit (CPU) resources. As shown in Figure 1, after a host CPU passes the experimental data to a GPU device and launches kernel functions on the GPU, the GPU runs independently and the CPU is free to continue communications with other hardware, so this can be very helpful for the applications where a fast data acquisition is needed. When the host program needs the data analyses results from the GPU, the CPU can communicate with the GPU again to transfer the result data from the global memory on the GPU to the hose memory when the GPU has completed the required computations.

### A Modified Maximum Likelihood Estimator for GPU-LMFit

It has been well-known that MLE is better in terms of precision than LSE in the data fitting analysis in both SRLM [3] and FLIM [4]. Laurence et al. [4] reported an efficient MLE formula 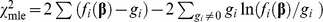, in which 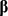 denotes the fitting parameters, 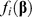 and 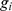 represent the model function and the experimental data, respectively, and the subscript 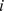 refers to the index of the data point. As with MPFit, GPU-LMFit is designed for directly using LSE in that it is to find a minimum of the sum of squares of the user-defined fitting function, so we simply modified Laurence’s MLE as follows:
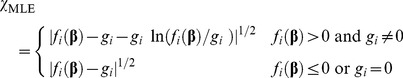
(6)where 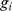 is required to be nonnegative and 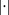 denotes absolute value. We used 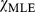 as the fitting function (i.e., 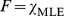 in Equation (1)) of GPU-LMFit to implement MLE fitting in both SRLM and FLIM.

### GPU2DGaussFit – A Massive Single-molecule Localization Program using GPU-LMFit in Superresolution Localization Microscopy

SRLM allows optical imaging in the far field beyond the diffraction limit and it dramatically improves the spatial resolution of optical microscopy to a few nanometers [26]. This technique relies on the precise localization measurements of single-molecules at the nanoscale. The localization analyses are generally performed by using model fitting technique to fit diffraction-limited single-molecule images with the theoretical PSF of the imaging system, which is commonly approximated as a two-dimensional (2D) Gaussian:
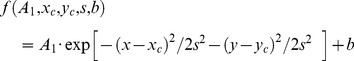
(7)where 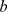 is the background offset, 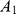 is the amplitude, 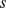 is the waist width and 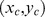 are the PSF center coordinates along 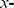 and 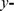
axis, respectively.

We have made an efficient program called GPU2DGaussFit to demonstrate how to use GPU-LMFit to fit massive single-molecule diffraction-limited images to a fitting function with Equations (6) and (7) for efficient localization analyses in SRLM. GPU2DGaussFit was coded with CUDA C/C++ code and compiled in Microsoft Visual Studio 2010 to a Matlab mex function, so GPU2DGaussFit can be called in Matlab to pass the experimental or simulated single-molecule images as arrays to the global memory of GPU, and then it launches three kernel functions in sequence to complete sets of 2D Gaussian fittings. The first kernel function is to initialize all fitting parameters in Equation (7) using a general method as follows: the maximum image intensity and the indices of the maximum intensity pixel are used as the initial values of the parameters 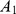 and 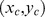 in a fit, respectively, while the initial value of the parameter 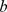 is the minimum image intensity and if the minimum image intensity is zero, a small intensity of 0.1 is added to each pixel of the image to avoid potential singular problems in optimization routine. The initial value of the parameter 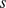 is used the true value which is used to generate the synthetic images in the simulation. The second kernel function is to call and execute GPU-LMFit. The fitting function is Equation (6) to use the modified Laurence’s MLE for best localization precision, and the model function 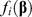 is Equation (7). The fitting function is implemented with parallel computing and it is passed to GPU-LMFit through a C-style pointer. The third kernel function is to correct the fitted parameter 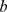 if the small intensity of 0.1 is added to the image by the first kernel function.

### GPUFLIMFit – an Efficient Fluorescence Lifetime Fitting Program using GPU-LMFit for Fluorescence Lifetime Imaging Microscopy

FLIM is a valuable and versatile tool for the investigation of the molecular environment of fluorophores in living cells by measuring the excited state lifetimes of interested fluorescence samples [5]. The lifetime of the fluorophore, rather than its fluorescence intensity, is used to create the image in FLIM, and this has the advantage of minimizing the effect of variations in fluorophore concentration, illumination intensity and photodynamic processes such as photobleaching, and visualizing the state of the environment around the fluorophore that affects the fluorophore lifetime properties. The TCSPC technique is a popular method to measure fluorescence lifetimes in the time domain. Using the TCSPC technique, fluorescence is excited by a high repetition rate pulsed laser. The arrival times of individual photons after laser pulses are recorded over many excitation and fluorescence cycles to create a TCSPC histogram. Fluorescence lifetime imaging using the TCSPC technique relies on the efficient and precise analysis of the lifetime estimation from the TCSPC histogram measured at each image pixel.

A widely used method is to use the model fitting technique [4] accomplished by a nonlinear model fitting procedure that involves iterative re-convolution of the IRF and single or multiple exponential functions. For example, a TCSPC histogram fitting program (Laurence’s software) with Laurence’s MLE formula 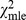 has been previously reported by Laurence and Chromy [4], and it can precisely fit TCSPC histogram to the following model:
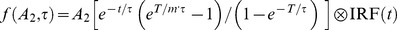
(8)


Here 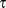 is the lifetime, 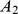 is the amplitude which is equal to the number of photons of the curve, 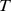 is the time window between laser pulses, and 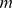 is the number of time bins in the time window 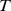. The time 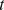 is calculated as 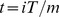, where 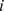 is integer from 1 to 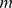, and represent the index of each time bin. The symbol 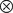 denotes convolution, which can be calculated efficiently using fast Fourier transform (FFT) algorithms as in Laurence et al [4]. Using GPU-LMFit, we made an efficient fluorescence lifetime fitting routine, called GPUFLIMFit, with a fitting function using Equations (6) and (8). Due to the lack of a GPU device callable FFT function in CUDA, the discrete convolution in Equation (8) is computed as Cauchy product [27] in parallel by all threads within a CUDA block. Laurence’s software is only executable on CPU and it was provided as a dynamic-link library (DLL) for Microsoft Windows operating systems and has an interface to LabVIEW (National Instruments, Austin, TX) so that it can be called from LabVIEW. Our GPUFLIMFit was also programed to have an interface to LabVIEW in order to compare the performance with Laurence’s software in the same computer system. In the simulations for the performance comparison of Laurence’s software and our GPUFLIMFit, the simulated TCSPC FLIM data were created in Matlab as described below, and then the data were read in LabVIEW to implement model fittings separately by using Laurence’s software and our GPUFLIMFit.

## Results

### The Performance of GPU2DGaussFit

GPU2DGaussFit uses GPU-LMFit to fit massive single-molecule images in SRLM to a 2D Gaussian. Previously, Smith et al. [3] reported an efficient GPU-based fitting software (Smith’s software) for SRLM, and importantly, Smith’s software can achieve the theoretical precision predicated by the Cramér-Rao lower bound (CRLB) approach [3] and ∼80-fold increase in speed by comparing with different minimization routines executed on CPU. Smith’s software is an ideal model program to which our GPU2DGaussFit can be compared for performance evaluation on the same GPU device, because the fitting speed and precision comparison between the programs running separately on GPU and CPU will depend not only on the algorithms used in the programs, but also on the hardware performance of the used GPU and CPU.

**Figure 3:**
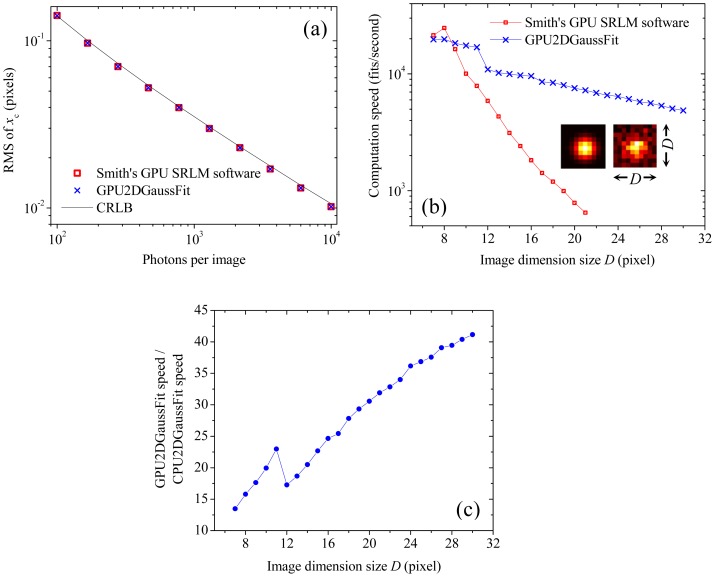
The performance of the implementation of GPU-LMFit in SRLM. (a) The model fitting precisions of our GPU2DGaussFit implemented with GPU-LMFit are compared with those of Smith’s GPU based SRLM software. Both GPU2DGaussFit and Smith’s software have nearly the same localization precisions, which are in excellent agreement with the theoretical predictions by the CRLB approach. Similar results were found for the *y*-Cartesian coordinates *y*_c_. (b) Our GPU2DGaussFit can work much more efficiently than Smith’s GPU based SRLM software to fit the relatively large size SRLM images to 2D Gaussian. Insets, a synthetic fluorescence single-molecule image (right) and its model (left) with a total of 100 photons in the simulation. (c) Computational speed comparison of GPU2DGaussFit and CPU2DGaussFit.

Both Smith’s software and our GPU2DGaussFit were built with a Matlab interface, so it is easy to conduct simulations in Matlab for the performance comparison between Smith’s software and GPU2DGaussFit. Figure 3 shows the results from two numerical simulations for the fitting speed and precision comparison between Smith’s software and GPU2DGaussFit, respectively. In the simulations, 5000 square single-molecule images were first numerically generated using the PSF function – Equation (7) and Poisson distributed shot noise was added using the function *poissrnd* from Matlab Statistics Toolbox. The synthetic images were passed into GPU memory, and then Smith’s software and GPU2DGaussFit were separately implemented on the same GPU device (NVIDIA Quadro 4000) to resolve the position of the single-molecule in each image. In Figure 3(a), the same parameters settings (i.e., 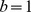, 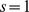 pixel, image size 7×7 pixels) as for Fig. 1 in Smith et al. [3] were used for the image generation, and total number of photons in each image was determined by 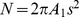. Smith’s software was set to output the theoretical localization precisions predicted by the CRLB approach [3] for both 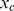 and 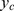 and fit the synthetic SRLM images to the PSF function with the parameter 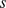 fixed. As shown in Figure 3(a), GPU2DGaussFit works as precisely as Smith’s software does, and it also achieves CRLB predicted precisions for the images over a wide range of signal to noise ratios (SNRs), so this can indicate that GPU-LMFit is a precise optimizer for SRLM. The other simulation for Figure 3(b) compares the fitting speed of both Smith’s software and GPU2DGaussFit. In this simulation, the size 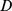 of each dimension of the synthetic square images varied as shown on the 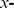
axis of Figure 3(b); other parameters for the synthetic images are 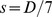 and 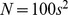, so that the SNRs and thus the resulting localization precisions (data not shown) of all images are the same as those of the images with 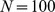 in Figure 3(a). In general, the more fitting parameters are free to be fit, the lower fitting speed is expected from an optimizer. Smith’s software in the simulation for Figure 3(b) was set to not output the CRLB precision but resolve all five parameters in the PSF function, which gives an equal number of fitting parameters in GPU2DGaussFit. Importantly, as shown in Figure 3(b), GPU2DGaussFit can perform faster than Smith’s software when the image size is larger than 10×10 pixels, and very intriguingly, GPU2DGaussFit can be more than 10× faster than Smith’s software for the images with more than 20×20 pixels, and this can indicate that the algorithms in GPU-LMFit has higher scalability and better parallel computing capability than those in Smith’s software.

We used MPFit to create a CPU version program called CPU2DGaussFit for 2D Gaussian function fittings in SRLM to compare the performance with GPU2DGaussFit. We included CPU2DGaussFit in the simulations in Figure 3. CPU2DGaussFit was implemented on an Intel eight-core CPU processor (W3550, 8M Cache, 3.06 GHz) and the computer has 16 gigabytes of physical memory. From the simulations, we have found that CPU2DGaussFit gives the same localization precisions (data not shown) for the images over the same range of SNRs in Figure 3(a) as GPU2DGaussFit does. Figure 3(c) shows that, as expected, GPU2DGaussFit can perform much faster than CPU2DGaussFit, especially for the synthetic single-molecule images with a larger dimension size. Therefore, we can conclude that the parallel LM algorithm in GPU-LMFit has much higher efficiency than the sequential LM algorithm in MPFit for the image in which each pixel contains large amount of independent data points involved in the fitting.

We have also validated GPU2DGaussFit with some previous experimental data [28] of single-molecule fluorescent quantum dots imaging with a total internal reflection fluorescence microscope. We compared the performance of GPU2DGaussFit in the localization analysis of the single-molecule quantum dots with a previously used Matlab program using the optimizer *fminunc* or *lsqnonlin* in the optimization toolbox in Matlab. From the comparison, we found that GPU2DGaussFit can perform as accurately as the Matlab program can, but not surprisingly, GPU2DGaussFit can run hundreds of times faster on the same computer used for Figure 3.

### The Performance of GPUFLIMFit

The fitting speed and precision of Laurence’s software and our GPUFLIMFit were compared in the numerical simulations, which were performed on simulated single exponential convolved with a Gaussian IRF:
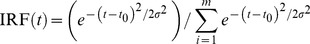
(9)where 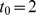 ns and 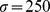 ps are used. In the simulations, 65,536 TCSPC histograms (corresponding to a 256×256 pixels TCSPC FLIM image) were created using Equations (8) and (9). In Equation (8), we took 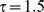 ns and 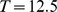 ns in the simulation. The simulated TCSPC histograms were added Poisson distributed shot noise using the function *poissrnd* in Matlab and saved to data files, which were then read in LabVIEW and passed to Laurence’s software and our GPUFLIMFit to resolve the fluorescence lifetimes and intensity amplitudes.

**Figure 4:**
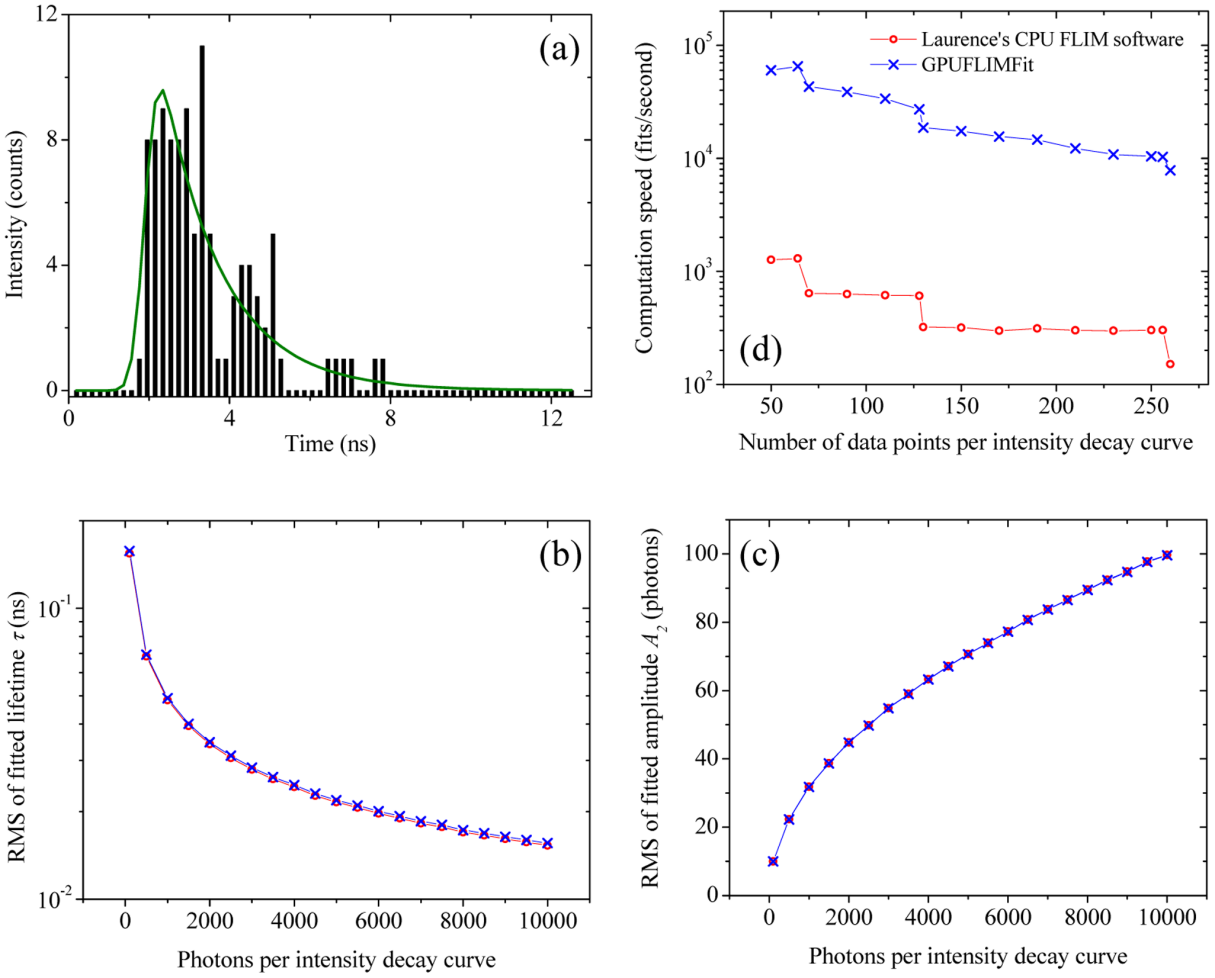
The performance of the implementation of GPU-LMFit in FLIM. (a) A synthetic TCSPC histogram (black bars) with a total of 102 photons and 64 data points, and its fitting curve (green line) from our GPUFLIMFit implemented with GPU-LMFit. (b-c) Both GPUFLIMFit and Laurence’s software show nearly identical fitting precisions for the fluorescence lifetimes 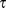 (b) and amplitudes 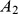 (c) in the model function, i.e., Equation (8). (d) Our GPUFLIMFit for TCSPC FLIM can work ∼49 times faster than Laurence’s software to fit the TCSPC histograms with different numbers of data points.

Figure 4 demonstrates that GPUFLIMFit implemented with GPU-LMFit accelerates the fluorescence lifetime fitting analysis in TCSPC FLIM. Figure 4(a) shows an example of fitting a synthetic TCSPC histogram with a total of 102 photons and 64 data points to the model – Equations (8) and (9). The first simulation for the fitting precision comparison was performed with the simulated 64 points TCSPC histograms in which the number of photons on each curve varies as indicated on the 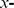
axis of Figures 4(b–c). Figures 4(b–c) show that both Laurence’s software and GPUFLIMFit give nearly identical precisions of the fitted lifetimes 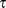 and amplitudes 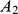 in this simulation, evidencing that GPU-LMFit can also be a precise optimizer for TCSPC FLIM. The other simulation for the fitting speed comparison in Figure 4(d) was performed with the simulated TCSPC histograms, each of which has 100 photons and a varying number of data points as indicated on the 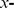
axis of the figure. In Figure 4(d), our GPUFLIMFit can perform averagely 49× faster than Laurence’s CPU software on the computer, in which the GPU device is a NVIDIA Quadro 4000 graphic card, and an Intel eight-core CPU processor (W3550, 8 M Cache, 3.06 GHz) and 16 Gbytes memory are installed.

**Figure 5:**
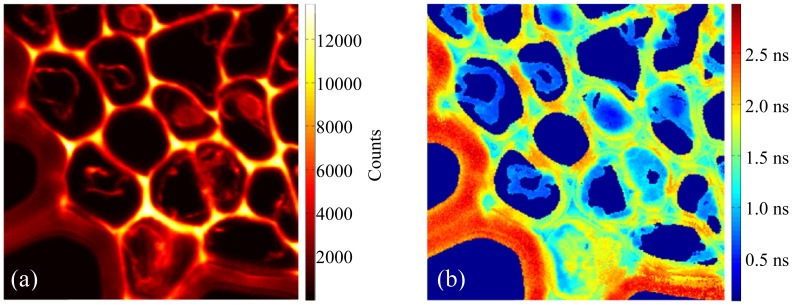
An example of fast automated experimental FLIM imaging using GPUFLIMFit. (a) An experimental confocal fluorescence intensity image of a cross-section of the rhizome of Convallaria taken with a multiphoton FLIM microscope. The image size is 40×40 µm^2^, 256×256 pixels. (b) The corresponding fluorescence lifetime image of (a) was generated in 0.6 seconds by using GPUFLIMFit implemented with GPU-LMFit.

Figure 5 shows an implementation of our GPUFLIMFit on the experimental FLIM imaging data of a cross-section of the rhizome of Convallaria (Lily of the Valley). The image data was taken using a multiphoton fluorescence confocal microscope which was developed based on an ISS Alpha FCS microscope (ISS, Champaign, IL) and upgraded with a TCSPC card and a two-photon excitation at 800 nm of the pulsed laser beam. Figure 5(a) is the fluorescence intensity image, which has 256×256 pixels and at each pixel a TCSPC histogram was measured with 64 channels (i.e., 64 data point per TCSPC histogram) by using a TCSPC card equipped with the microscope. Using GPUFLIMFit, the generation of the fluorescence lifetime image in Figure 5(b) was accomplished in ∼0.6 seconds. An intensity threshold of 400 counts was used in the calculation of the lifetimes so that a total of 42834 pixels were involved in the calculation.

### Limitations of the Study, Open Questions, and Future Work

The LM algorithm is an iterative procedure. Before the procedure can begin, one needs to estimate an initial value for each fitting parameter. For a complete new model, if one cannot be sure whether the initial values are correct, it will require the user to graph the curve defined by some estimated initial values. If the resulting curve comes close to the data, the parameter values can be chosen to start the fitting process. However, for the massive parallel model fitting processes in GPU-LMFit, it is necessary that the program can automatically compute the initial values. In our two application examples in SRLM and FLIM, the initial estimates of some fitting parameters including 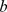, 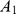, 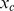 and 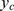 in Equation (7) as well as 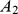 in Equation (8) can be calculated from the experimental data; other fitting parameters have physically limited small value ranges – the parameter 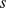 in Equation (7) ranges in practice from half a pixel to a few pixels and the parameter 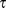 in Equation (8) is generally from hundreds of picoseconds to ∼10 ns. The LM algorithm has been proved very robust and stable, and we found that it can work well if the parameter 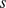 or 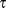 is simply initialized with a constant value in its physical value range.

GPU2DGaussFit fits massive single-molecule images to Equation (7) to retrieve the positions of single-molecules, and in SRLM this is a conventional single-molecule fitting method, which is known not as accurate for high molecular density SRLM images as for low molecular density images. This is because the single-molecule signals can overlap in a high molecular density image, and Equation (7) cannot be the good model for this type of images. A number of methods [29] have been reported to be suitable for high molecular density images, but they are usually found to be less accurate for low molecular density image than single-molecule fitting methods.

Astigmatic imaging [26] is one of several existing methods for three-dimensional (3D) SRLM. It is achieved by adding a cylindrical lens to the imaging path to make the axial symmetry of the PSF image of each emitter dependent on its axial position. The 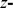
coordinate for each emitter can be retrieved by using a predetermined astigmatic calibration curve of the optical system. It has been reported [30] that fast anisotropic 2D Gaussian fitting can accelerate the 3D post-acquisition reconstruction analysis in 3D SRLM using astigmatic imaging. Following the method in [30], some simple modifications can also be made on GPU2DGaussFit to enable the use of GPU-LMFit for fast 3D astigmatic imaging. For example, the fitting function, i.e., Equation (7), should be replace with an anisotropic 2D Gaussian.

For most applications in SRLM and FLIM, both GPU2DGaussFit and GPUFLIMFit can allow the generation of parametric imaging microscopy images much faster than the image data acquisition, so the model fitting speed is generally to be sufficient for real-time data analysis. As discussed in Figure 1, the architecture of GPU-LMFit is also suitable to directly use multiple GPUs to further increase the efficiency of GPU-LMFit for the applications with the continuously increased rate of data output in new microscopy methods using new detector technologies.

A variety of methods have been developed to extract the single-molecule positions from SRLM data [3], [29], [31] and the lifetimes from FLIM data [4], [32], [33]. Some [29], [33] of these methods are not model fitting methods, and the performance comparison of the different methods is out of the scope of this paper, while others [4], [31] compare only the different fitting model functions. In this work, we focus on the performance optimization of the LM optimizer and provide GPU-LMFit to achieve efficient and accurate massive parallel model fitting computations for real-time automated parametric imaging microscopy.

## Conclusions

We applied high performance scalable parallel computing technique based on GPU implementation to the LM algorithm and provide an accurate, robust and very efficient minimization optimizer, GPU-LMFit. We demonstrate the performance of GPU-LMFit in the massive model fitting applications in SRLM and FLIM, and found that GPU-LMFit is an efficient optimizer that can enable real-time automated pixel-wise parametric imaging microscopy. It can be concluded that GPU-LMFit should be directly useful in a wide variety of automated pixel-wise parametric imaging microscopy techniques and many other fields where experimental data analyses rely on model fitting techniques.

## Supporting Information

File S1 **Supplementary Software.** The complete package includes a user’s manual, the 32-bit CUDA C libraries of GPU-LMFit, the example source code of GPU2DGaussFit and the Matlab simulation programs for the performance tests of both GPU2DGaussFit and GPUFLIMFit.
(ZIP)
