# Supplementary material for: Efficient Parallel Levenberg-Marquardt Model Fitting towards Real-Time Automated Parametric Imaging Microscopy
Source: PLoS One. 2013 Oct 10;8(10):e76665. doi: 10.1371/journal.pone.0076665 (PMC3794933; doi:10.1371/journal.pone.0076665)
Supplement: File S1 — Supplementary Software. The complete package includes a user’s manual, the 32-bit CUDA C libraries of GPU-LMFit, the example source code of GPU2DGaussFit and the Matlab simulation programs for the performance tests of both GPU2DGaussFit and GPUFLIMFit. (ZIP) [file pone.0076665.s001.zip › Manual.pdf]

GPU-LMFit solution

Xiang Zhu and Dianwen Zhang

August 2013

1. Introduction

This mini-manual of GPU-LMFit introduces how to setup your computer system to use the GPU-LMFit library in Visual Studio 2010 (VS2010, http://www.microsoft.com). Here we take the C++ project ***GPU2DGaussFit*** as an example to use GPU-LMFit for massive single-molecule localization analyses in superresolution localization microscopy (SRLM). In the example, GPU2DGaussFit is created with a Matlab (http:// www.mathworks.com) interface, and the fitting function is either

$$\chi_{\mathrm{LSE}} = f_i(\boldsymbol{\beta}) - g_i \tag{1}$$

for least-squares estimator (LSE) or

$$\chi_{\mathrm{MLE}} = \begin{cases} \left| f_i(\boldsymbol{\beta}) - g_i - g_i \ln\left(f_i(\boldsymbol{\beta})/g_i \right) \right|^{1/2} & f_i(\boldsymbol{\beta}) > 0 \text{ and } g_i \neq 0 \\ \left| f_i(\boldsymbol{\beta}) - g_i \right|^{1/2} & f_i(\boldsymbol{\beta}) \leq 0 \text{ or } g_i = 0 \end{cases} \tag{2}$$

for the modified Laurence's maximum likelihood estimator (MLE)[1], where $\boldsymbol{\beta}$, $f_i(\boldsymbol{\beta})$ and g_i denote the fitting parameters, the model function and the experimental data, respectively. The model function f_{SRLM} for GPU2DGaussFit is a general form of a two-dimensional (2D) Gaussian:

$$f_{\mathrm{SRLM}}(A_1, x_c, y_c, s, bg_1) = A_1 \cdot \exp\left[-(x - x_c)^2 / 2s^2 - (y - y_c)^2 / 2s^2 \right] + bg_1 \tag{3}$$

to approximate the point spread function (PSF) of the imaging system in localization microscopy, where bg_1 is the background offset, A_1 is the amplitude, s is the waist width and (x_c, y_c) are the PSF center coordinates along x- and y-axis, respectively.

The C code VS2010 project of GPU2DGaussFit is located in the folder ".\GPU2DGaussFit", and after compilation with default settings, a Matlab (The Mathworks, Natick, USA) mex function **GPU2DGaussFit.mexw32** is created and stored in the folder ".\GPU2DGaussFit Test", where two Matlab m-script files **Test_GPU2DGaussFit_Accuracy.m** and **Test_GPU2DGaussFit_Speed.m** are provided to test the accuracy and speed of the program GPU2DGaussFit.mexw32, respectively.

The Matlab mex function **GPUFLIMFit.mexw32** included with this software package is an efficient time-correlated single-photon counting (TCSPC) histogram fitting program using GPU-LMFit for TCSPC fluorescence lifetime imaging microscopy (FLIM), and it is located in the folder ".\GPUFLIMFit Test", where a Matlab m-script file **Test_GPUFLIMFit.m** can also be found for testing the performance of the program GPUFLIMFit.mexw32. The fitting model function f_{FLIM} for GPUFLIMFit is given as follows:

$$f_{\text{FLIM}}\left(A_{\text{SHG}}, A_2, \tau, bg_2\right) = bg_2 + A_{\text{SHG}}\delta\left(t - T/m\right) + A_2\left[e^{-t/\tau}\left(e^{T/m\cdot\tau} - 1\right)\Big/\left(1 - e^{-T/\tau}\right)\right] * \text{IRF}\left(t\right) \quad (4)$$

Here bg_2 is the background offset, A_{SHG} is the amplitude of second harmonic generation (SHG), $\delta()$ is the Dirac delta function, A_2 is the amplitude which is equal to the number of photons of the curve, τ is the lifetime, T is the time window between laser pulses, and m is the number of time bins in the time window T. The time t is calculated as $t = iT/m$, where i is an integer from 1 to m and represents the index of each time bin. The symbol $*$ denotes convolution, which is computed as Cauchy product[2] in parallel by all threads within a CUDA block.

Both GPU2DGaussFit.mexw32 and GPUFLIMFit.mexw32 in this software package are for 32 bit Matlab. 64 bits programs may also be possible to create or would probably already been available for certain platforms upon request.

2. System requirements

The implementation of GPU-LMFit requires a PC with a CUDA (http://www.nvidia.com) capable graphics card. GPU2DGaussFit and GPUFLIMFit were tested on a computer in which the GPU device is an NVIDIA Quadro 4000 graphic card, and an Intel eight-core CPU processor (W3550, 8M Cache, 3.06 GHz) and 16 Gbytes memory are equipped. The tests were done on Window 7 64-bit system with Visual Studio 2010, Matlab 2011B x86 and CUDA 5.5 (64-bit). The recommended order of the installation of these software products is to install Visual Studio 2010 first, followed by Matlab 2011B x86, and lastly CUDA 5.5 (64-bit).

64-bit version GPU-LMFit is not included in the supplementary software package, but it has been preliminarily tested by using Visual Studio 2010 with the native 64-bit "AMD64" compiler and Matlab 2011B (64-bit) and CUDA 5.5 (64-bit). This version of 64-bit GPU-LMFit is available upon request.

To use the below GPU-LMFit library, separate compilation in CUDA is required. Prior to the 5.0 release, CUDA did not support separate compilation, so all device code needs to be within one *.cu file, and could not call device functions or access variables across files. According to the manual of CUDA compiler driver nvcc, separate compilation only works for

the GPU devices with compute capability 2.0 and above, and thus a GPU device of compute capability >2.0 is required to use the GPU-LMFit library.

3. GPU-LMFit library files

GPU-LMFit is made using CUDA C/C++ language, and its source code can be available upon request or in public domain after our publication. In this software package, GPU-LMFit is made to the standard C-style library functions (located in the folder ".\GPU-LMFit\") and it includes four files:

GPU_LMFit_Win32.lib contains the definitions of some variables and the main function GPU_LMFit() which is CUDA device callable function, and should be called from a user's kernel function for the implementation of the massive parallel Levenberg-Marquardt model fittings on GPUs. It supports programming on both 32-bit and 64-bit versions of Windows operating systems. The 64-bit version (GPU_LMFit_x64.lib) of GPU-LMFit library is also available for certain platforms upon request.

GPU_LMFit.cuh is a header file providing the application programming interface (API) of the library GPU_LMFit_Win32.lib (or GPU_LMFit_x64.lib). It defines types and macros and declares variables and the prototype of the function GPU_LMFit(). It needs to be included in the user's project for using GPU-LMFit.

GPU_LMFit_Accessories_Win32.lib is an accessory library of the 32-bit GPU-LMFit library (GPU_LMFit_Win32.lib). It is supplied with the functions only callable from the host to help users compute the smallest size of the global/shared memory required for temporarily storing work variables in GPU-LMFit on a GPU device. A 64-bit accessory library GPU_LMFit_Accessories_x64.lib will be required for the 64-bit GPU-LMFit library GPU_LMFit_x64.lib.

GPU_LMFit_Accessories.h is a header file providing the application programming interface (API) of the library GPU_LMFit_Accessories_Win32.lib (or GPU_LMFit_Accessories_x64.lib), and it also needs to be included in the user's project to help users program host code to allocate enough buffer memory on GPU devices for running GPU-LMFit.

4. Using GPU-LMFit library in Visual Studio 2010 to build the project – GPU2DGaussFit

Here we demonstrate how to build the project GPU2DGaussFit using the 32-bit GPU-LMFit library (GPU_LMFit_Win32.lib) from creating a new project in Visual Studio 2010 by the following steps:

1) In an empty solution of Visual Studio 2010, click on the menu "File → New → Project…", or in an existing solution, click on the menu "File → Add → New Project…";

2) Select "Win32 Project" and enter a project name, which is GPU2DGaussFit in this example, and click on "OK" and then "Next";
3) Select "DLL" for the application type and "Empty Project" in the additional options, and click on "Finish";
4) In the "Solution Explorer" panel, right click on the project name, and select "Build Customization…" and then select the build customization files for the installed version of CUDA. In our case, it is "CUDA 5.5 (.targets, .props)".
5) Locate three folders – gpulmfitlibroot, matlabroot and cudaroot, which represent, respectively, the names of the folders where the GPU-LMFit library, MATLAB and CUDA software are installed. For example, matlabroot = "C:\Program Files (x86)\MATLAB\R2011b" and cudaroot = "C:\Program Files\NVIDIA GPU Computing Toolkit\CUDA\v5.5" in our case. Please note that the locations and names of the folders for Matlab and CUDA are subject to change for different system. For the GPU-LMFit library, the header and library files are respectively stored in the subfolders "include" and "lib" of gpulmfitlibroot.
6) Set "Build → Configuration Manager → Active solution configuration" to "Release" for optimal performance of the target project.
7) In the "Solution Explorer" panel, right click on the project name, select "Properties → Configuration Properties" and complete the following steps:
    a. Change "General → Target Extension" to be ".mexw32" for our case, or ".mexw64" for a 64-bit project.
    b. Add the folder "cudaroot" to "CUDA C/C++ → Common → CUDA Toolkit Custom Dir";
    c. Add "gpulmfitlibroot\include" and "matlabroot\extern\include" to "CUDA C/C++ → Common → Additional Include Directories";
    d. Choose "Yes (-rdc=true)" for the option "CUDA C/C++ → Common → Generate Relocatable Device Code";
    e. For our case, choose "32-bit (--machine 32)" for the option "Target Machine Platform", or if one compiles a 64-bit project, "64-bit (--machine 64)" should be chosen for this option;
    f. Specify the compute capability of the GPU(s) by adding, for example, "compute_20,sm_20" for our NVIDIA Quadro 4000 graphic card to "CUDA C/C++ → Device → Code Generation";
    g. Add "gpulmfitlibroot\lib\$(Platform)" [1], "matlabroot\extern\lib\$(Platform)", and "cudaroot\lib\$(Platform)" to "Linker → General → Additional Library Directories";
    h. Add "libmx.lib", "libmex.lib", "libmat.lib", "cudart.lib", "cuda.lib", "GPU_LMFit_Accessories_$(Platform).lib" and "CPU_LMFit_$(Platform).lib" to "Linker → Input → Additional Dependencies".
    i. Add "cudaroot\lib\$(Platform)" to "CUDA Linker → General → Additional Library Directories".

[1] $(Platform) is a macro definition in Visual Studio 2010 and it represents "Win32" for 32-bit compilation or "x64" for 64-bit compilation

j. Add “CPU_LMFit_$(Platform).lib” to “CUDA Linker → General → Additional Dependencies”.

8) Add a module-definition (.def) file (it is “Apps.def” in this example project) to “Linker → Input → Module Definition File”; the module-definition file provides the linker with information about exports, attributes, and other information about the dynamic-link library (DLL) for the application to be linked.
9) Create and edit all *.cuh, *.cu, and *.def files (the main program file is “Apps.cu” in this example project).
10) Right click on each *.cu file, and set “Properties → General → Item Type” to “CUDA C\C++”.
11) Right click on each *.cuh and *.def file, and set “Properties → General → Item Type” to “Does not participate in build”.
12) Compile and build the project.

5. GPU2DGaussFit

32-bit GPU2DGaussFit.mexw32 can be called directly in Matlab use the following syntax:

x = GPU2DGaussFit(***ImgsData***, ***ImgDimension***, ***Init_s***, ***FitMethod***, ***JacMethod***, ***GPU_device_ID***, ***GPU_Block_Size***, ***GPU_Grid_Size***);

or [***x infonum***] = GPU2DGaussFit(***ImgsData***, ***ImgDimension***, ***Init_s***, ***FitMethod***, ***JacMethod***, ***GPU_device_ID***, ***GPU_Block_Size***, ***GPU_Grid_Size***).

The parameters are described below:

ImgsData is square image data array in single-precision data type. If it is originally two-dimensional, it need to be converted to one-dimensional.

ImgDimension is the dimension size of the images. It must be a scalar.

Init_s is the initial value (Default = 1.0f pixel) of the Gaussian waist width.

FitMethod is to select fitting methods (estimators): 0 – maximum likelihood estimator (MLE) or 1 – unweighted least squares (uWLS);

JacMethod is to select the method for computing Jacobian matrix. It is zero to use analytical Jacobian matrix (Note: no analytical Jacobian function is available for MLE in this program), or otherwise numerical approximation to Jacobian matrix will be calculated in GPU-LMFit.

GPU_device_ID is the device number of GPU to be used if multiple GPU devices are available. Or it is zero if only one GPU is in the system;

GPU_Block_Size and ***GPU_Grid_Size*** are for user to preset the maximum block size and the maximum grid size of CUDA, respectively;

CUDA block size can affect the computational efficiency of this program. Obviously too small block size can limit the degree of parallelism and won't allow the best parallel result, but too large block size can also cause the negative effects on the performance of this program. For example, all threads share the limited resources (e.g., registers, shared memory and *etc*.) on GPU, so too many threads in a CUDA block can decrease the number of concurrent blocks, and as a result, they may increase rather than decrease the amount of time required to finish all tasks. To avoid this effect, the parameter GPU_Block_Size allows user to specify a maximum number of threads per block on GPU.

Now CUDA and GPU devices can allow a large grid size, for example, a grid with 65535 blocks can be specified for CUDA capability 2.0 at run time, but the computation time for a kernel function with too many blocks on GPU can be too long, and it can activate a timeout detection and recovery (TDR) process in Windows operation system, and windows can automatically restart/reset a GPU graphic device, so the computation will be unexpectedly stopped and consequently all results will also be lost. To avoid this problem, the parameter GPU_Grid_Size allows user to specify a smaller maximum number of blocks for each call of the kernel functions, which are running on GPU, and the program completes the entire tasks through multiple iterative calls of the kernel functions. The problem can also be avoided by modifying several windows TDR-related registry keys (For more details about this solution, users can refer to the Microsoft online article "Timeout Detection and Recovery of GPUs" at http://msdn.microsoft.com/en-us/library/windows/hardware/ff570088(v=vs.85).aspx).

x is fitted parameters, ***infonum*** can be the Chi square of each fit or the exit codes of GPU-LMFit depending on the setting of a conditional compilation constant _RETURN_INFONUM_ in the project.

6. GPUFLIMFit

32-bit GPUFLIMFit.mexw32 can be called directly in Matlab use the following syntax:

[***x Chisq fval***] = FLIMFit(***DecayData***, ***IRF***, ***DecayT***, ***InitPS***, ***UseBkOffset***, ***UseSHGAmp***, ***FitMethod***, ***NumOfExps***, ***UseLBs***, ***LBs***, ***UseUBs***, ***UBs***, ***GPU_Device_ID***, ***GPU_Block_Size***, ***GPU_Grid_Size***)

The parameters are described below:

DecayData must be in single-precision floating-point format. It is one-dimensional data array that contains the data for multiple TCSPC histogram curves, which are organized sequentially.

IRF must be in single-precision floating-point format. It is data array for the instrument response function, and it can have two formats:

1) It has the same number of data point as that of each TCSPC histogram curve.
2) It has the same size in computer memory as the first input parameter DecayData for the case that each TCSPC histogram curve has its own IRF.

DecayT must be in single-precision floating-point format. It is the time t values on x-axis in TCSPC histogram, and it must be the same size as a single TCSPC histogram.

InitPS must be in single-precision floating-point format. It contains the initial values of the fitting parameters of the fitting model and it has different data structures for different settings of the below two parameters UseBkOffset and UseSHGAmp. See the below description for the input parameter FitMethod.

UseBkOffset = 0, no background offset bg_2 in the fitting model. Or, the background offset bg_2 is introduced.

UseSHGAmp = 0, not includes the parameter A_{SHG} for the amplitude of second harmonic generation (SHG) in the fitting model. Or, the SHG amplitude is included.

FitMethod must be a 32 bits integer data format. It is to select for fitting methods. It is zero for MLE fitting, or 1 for LSE. The parameter InitPS should be organized as follows:

1) UseBkOffset = 0 && UseSHGAmp = 0
   InitPS = [$A_2(i,1)$, $A_2(i,2)$, ..., $A_2(i,n)$, $\tau(i,1)$, $\tau(i,2)$, ..., $\tau(i,n)$, ...].
2) UseBkOffset = 1 && UseSHGAmp = 0
   InitPS = [$bg_2(i)$, $A_2(i,1)$, $A_2(i,2)$, ..., $A_2(i,n)$, $\tau(i,1)$, $\tau(i,2)$, ..., $\tau(i,n)$, ...].
3) UseBkOffset = 0 && UseSHGAmp = 1
   InitPS = [$A_{SHG}(i)$, $A_2(i,1)$, $A_2(i,2)$, ..., $A_2(i,n)$, $\tau(i,1)$, $\tau(i,2)$, ..., $\tau(i,n)$, ...].
4) UseBkOffset = 1 && UseSHGAmp = 1
   InitPS = [$bg_2(i)$, $A_{SHG}(i)$, $A_2(i,1)$, $A_2(i,2)$, ..., $A_2(i,n)$, $\tau(i,1)$, $\tau(i,2)$, ..., $\tau(i,n)$, ...].

Here, $bg_2(i)$, $A_{SHG}(i)$, $A_2(i,j)$ and $\tau(i,j)$ represent, respectively, the values of the background offset, the SHG amplitude, and the fluorescence amplitude and lifetime of the j-th exponentials for the i-th TCSPC histogram. n and m is the total numbers of exponentials and TCSPC histograms, respectively.

NumOfExps must be a 32 bits integer data format. It is the number of exponential functions in the fitting model.

UseLBs must be a 32 bits integer data format. It is a scalar to disable the lower bounds LBs of all fitting parameters, or it is a vector in which zero-value elements disable specific lower bounds in LBs or nonzero-values enable.

LBs must be in single-precision floating-point format. It is a vector of the lower bounds of the fitting parameters.

UseUBs must be a 32 bits integer data format. It is a scalar to disable the upper bounds UBs of all fitting parameters, or it is a vector in which zero-value elements disable specific upper bounds in UBs or nonzero-values enable.

UBs must be in single-precision floating-point format. It is a vector of the upper bounds of the fitting parameters.

GPU_device_ID, ***GPU_Block_Size*** and ***GPU_Grid_Size*** have the same function and requirements for settings as those for GPU2DGaussFit.

x is a vector for all fitted parameters, ***Chisq*** is a vector for the Chi square value of each fit, and ***fval*** is the value of the fitting model function at the solution ***x***.

References

1. Ted A. Laurence and Brett A. Chromy, *Nat Meth* **7** (5), 338 (2010).
2. T.M. Apostol, *Modular Functions and Dirichlet Series in Number Theory*. (Springer Verlag, 2012).
