# Supplementary material for: Efficient Parallel Levenberg-Marquardt Model Fitting towards Real-Time Automated Parametric Imaging Microscopy
Source: PLoS One. 2013 Oct 10;8(10):e76665. doi: 10.1371/journal.pone.0076665 (PMC3794933; doi:10.1371/journal.pone.0076665)

One output from Test_GPUFLIMFit.m

Total number of points on each curve is 64...

True values: Amp = 400, Tau = 2.5

Generate Noised Decay Curves ... Total photons = 397...

... MLE individual fit ... Averaging 69943 fit per seconds ...

... Statistical analysis of fitting results ...

mean(MLE0_Taus-Tau) = 0.00250647

std(MLE0_Taus) = 0.147361

mean(MLE0_Amps) = 400.192

std(MLE0_Amps) = 20.0805

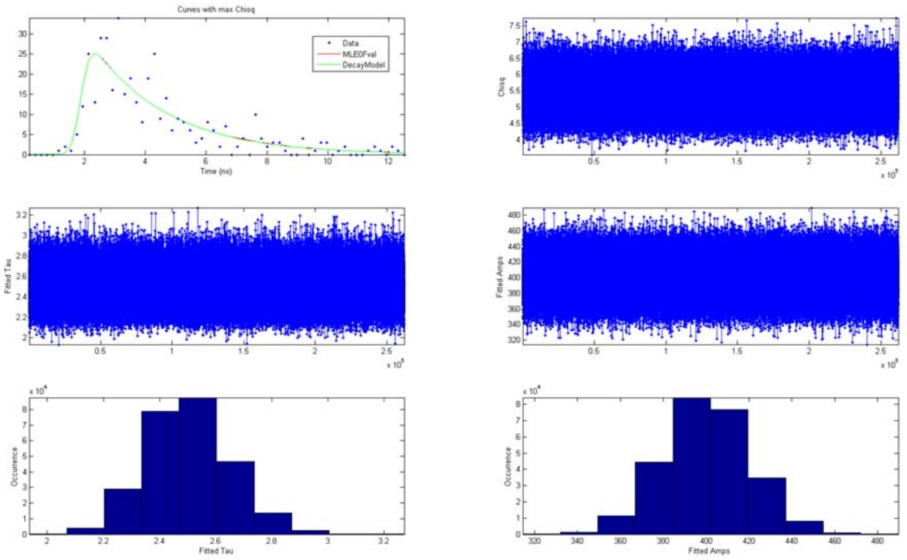

Supplement: File S1 — Supplementary Software. The complete package includes a user’s manual, the 32-bit CUDA C libraries of GPU-LMFit, the example source code of GPU2DGaussFit and the Matlab simulation programs for the performance tests of both GPU2DGaussFit and GPUFLIMFit. (ZIP) [file pone.0076665.s001.zip › GPUFLIMFit Test/One Test_GPUFLIMFit output.pdf]
