# Supplementary figures and images for: Efficient Parallel Levenberg-Marquardt Model Fitting towards Real-Time Automated Parametric Imaging Microscopy
Source: PLoS One. 2013 Oct 10;8(10):e76665. doi: 10.1371/journal.pone.0076665 (PMC3794933; doi:10.1371/journal.pone.0076665)

One output from Test\_GPU2DGaussFit\_Accuracy.m

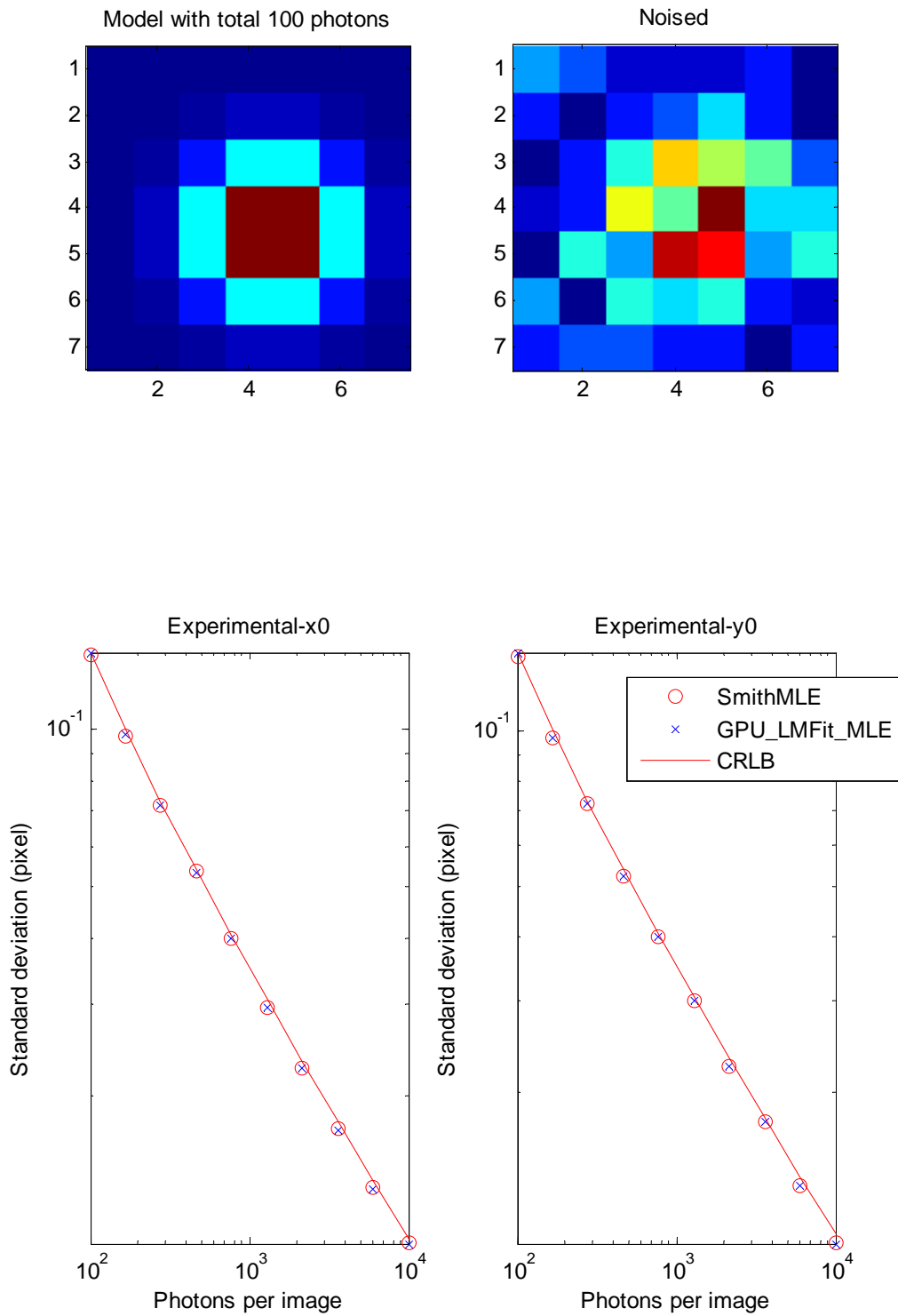

Supplement: File S1 — Supplementary Software. The complete package includes a user’s manual, the 32-bit CUDA C libraries of GPU-LMFit, the example source code of GPU2DGaussFit and the Matlab simulation programs for the performance tests of both GPU2DGaussFit and GPUFLIMFit. (ZIP) [file pone.0076665.s001.zip › GPU2DGaussFit Test/One Test_GPU2DGaussFit_Accuracy output.pdf]

One output from Test\_GPU2DGaussFit\_Speed.m

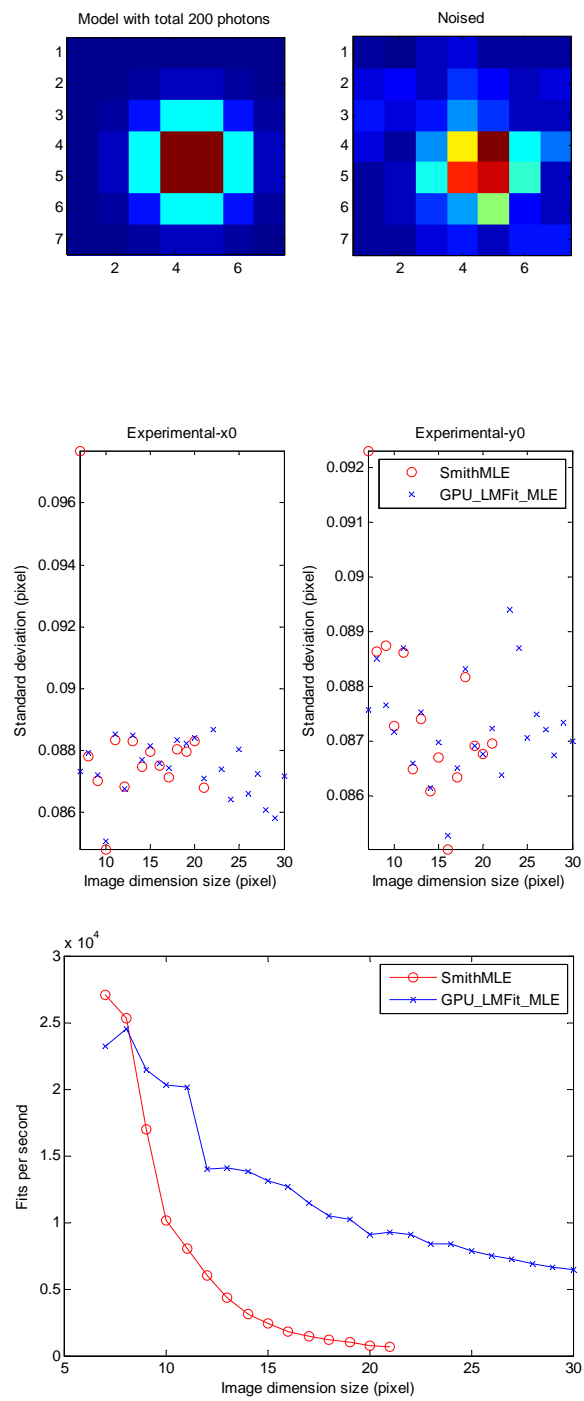

Supplement: File S1 — Supplementary Software. The complete package includes a user’s manual, the 32-bit CUDA C libraries of GPU-LMFit, the example source code of GPU2DGaussFit and the Matlab simulation programs for the performance tests of both GPU2DGaussFit and GPUFLIMFit. (ZIP) [file pone.0076665.s001.zip › GPU2DGaussFit Test/One Test_GPU2DGaussFit_Speed output.pdf]
